# Proteins from Legumes, Cereals, and Pseudo-Cereals: Composition, Modification, Bioactivities, and Applications

**DOI:** 10.3390/foods13131974

**Published:** 2024-06-22

**Authors:** Wenxue Zhang, Isaac Duah Boateng, Jinsheng Xu, Yi Zhang

**Affiliations:** 1 Department of Food Science, The Pennsylvania State University, University Park, PA 16802, USA; wmz5152@psu.edu; 2 Certified Group, 199 W Rhapsody Dr, San Antonio, TX 78216, USA; boatengisaacduah@gmail.com; 3 Hubei Key Laboratory of Agricultural Bioinformatics, College of Informatics, Huazhong Agricultural University, Wuhan 430070, China; jsxu@webmail.hzau.edu.cn

**Keywords:** plant protein, soy, cereal, pseudo-cereal, protein modification

## Abstract

This review presents a comprehensive analysis of plant-based proteins from soybeans, pulses, cereals, and pseudo-cereals by examining their structural properties, modification techniques, bioactivities, and applicability in food systems. It addresses the critical need for a proper utilization strategy of proteins from various plant sources amidst the rising environmental footprint of animal protein production. The inherent composition diversity among plant proteins, their nutritional profiles, digestibility, environmental impacts, and consumer acceptance are compared. The innovative modification techniques to enhance the functional properties of plant proteins are also discussed. The review also investigates the bioactive properties of plant proteins, including their antioxidant, antimicrobial, and antitumoral activities, and their role in developing meat analogs, dairy alternatives, baked goods, and 3D-printed foods. It underscores the consideration parameters of using plant proteins as sustainable, nutritious, and functional ingredients and advocates for research to overcome sensory and functional challenges for improved consumer acceptance and marketability.

## 1. Introduction

The United Nations predicts a global population rise to 10 billion by 2050 [1], which along with rising incomes and urbanization will lead to major shifts in food consumption patterns. It necessitates a change in both the quantity and variety of dietary foods [2]. Proteins, essential for human health due to their multifaceted functions, including catalyzing biochemical reactions, transporting essential substances, maintaining metabolic processes, providing energy, and facilitating cellular communication [3], are at the forefront of this demand shift. The global protein demand is expected to double by 2050, underscoring the need to evaluate food proteins.

Currently, dietary proteins are primarily sourced from animals, which include red meat, poultry, dairy, eggs, and seafood. Animal-based foods, however, contribute substantially to greenhouse gas (GHG) emissions, impacting climate change [1]. The demand for animal-based proteins, if it is escalated, will further exert pressure on land resources due to more feed cultivation and lead to the conversion of natural habitats into agricultural land, thereby exacerbating GHG emissions and biodiversity loss [4]. Therefore, it is urgent to explore sustainable alternatives, particularly plant-based proteins, to meet global protein needs responsibly.

Plant-based proteins present in foods like grains, legumes, nuts, tea, and pseudo-cereals are increasingly recognized for their dietary value [2]. Proteins from soybeans have been used widely in various food products and as a staple in vegan diets. Soy protein is a complete protein source, offering all essential amino acids as well as bioactive compounds, such as phytoestrogens and polyphenols that potentially reduce blood cholesterol levels and impede the proliferation of cancer cells [5]. Despite its benefits, popularity, and growing production, soy protein poses a few challenges to consumer acceptance due to undesirable functionalities and sensory attributes as well as allergenic potential [6]. Therefore, there is an ongoing need to explore a broader range of plant-based protein sources.

Recent research has focused on alternative plant-based proteins from pulses and cereal grains. These crops provide over half of the total caloric intake globally and are a major protein source owing to their affordability and nutritional benefits [7]. Pulses, including lentils, beans, chickpeas, and peas, are rich in protein, fiber, vitamins, and minerals, while low in fat. Their consumption is associated with health promotion, such as reduced low-density lipoprotein (LDL) cholesterol levels, and reduced risks of heart disease, type-2 diabetes, and colon cancer [8]. Certain cereals, such as rice and oats, are noted for their low allergenicity and high digestibility, making them suitable for dairy alternatives, protein snacks, and meat substitutes [9]. Pseudo-cereals, such as amaranth, quinoa, and buckwheat, have attracted attention due to their nutritional value and suitability for individuals with celiac disease [9,10]. Thus, pulses and cereals are increasingly viewed as viable alternative protein sources.

**Figure 1:**
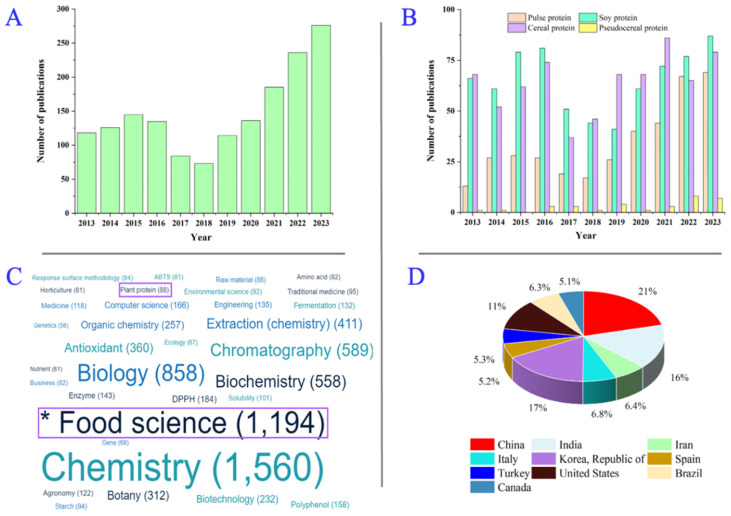
Scholarly works on plant-based protein. (**A**) Number of publications from 2013 to 2023. (**B**) Number of publications on proteins from pulses, soybeans, cereals, and pseudo-cereals. (**C**) Fields of studies. (**D**) Countries active in studies. (Data were taken from Lens.org on 1 May 2024).

Numerous review articles have highlighted the extraction, modification, and functional properties of pulse, cereal, and soy proteins. Despite this, it is imperative to acknowledge the inherent diversity among plant proteins. The difference is exhibited in nutritional profiles, digestibility, bioavailability, environmental impacts, consumer acceptance, and functional characteristics [8]. However, a systematic comparison of plant proteins remains unexplored in literature. This review addresses this gap by examining the structural composition, diverse modification techniques, bioactivities, and potential applications of various plant proteins. The objective is to underscore the viability and potential of plant proteins as sustainable alternatives for human nutrition and global protein supply. Figure 1 provides a detailed analysis of plant proteins through a comprehensive literature survey with data sourced from Lens.org databases. There is an increasing trend in the number of publications regarding plant-based proteins, especially from 2018 to 2023 (Figure 1A). Soy protein has been the most popular subject, followed by cereal protein and pulse protein (Figure 1B). Research on plant-based proteins is mainly from food science and chemistry disciplines (Figure 1C), and reported by researchers in China (21%), Republic of Korea (17%), India (16%), and the United States (11%) (Figure 1D).

## 2. Structural Composition of Soy, Pulse, Cereal, and Pseudo-Cereal Proteins

Proteins sourced from soybeans, pulses, cereals, and pseudo-cereals vary in composition and functionality. In a biological context, soybeans and pulses belong to the *Fabaceae* family (legumes); cereals are classified under the *Poaceae* family (grasses); and pseudo-cereals are from different botanical families and are grouped due to their grain-like characteristics. Thus, it is essential to compare soy proteins versus pulse proteins, as well as cereal proteins versus pseudo-cereal proteins. 

The main components of soybeans, pulses, cereals, and pseudo-cereals differ (Table 1), making them adaptable for a range of dietary requirements and preferences. Soybeans are notable for a high protein content, ranging between approximately 35% to 55%. Soy proteins offer a complete amino acid profile, making them an excellent plant-based protein [11]. Pulse crops, including peas, chickpeas, beans, and lentils, although containing a lower protein level (20–30%) compared to soybeans, provide a balanced mix of protein and dietary fiber. This combination is beneficial for digestive health and weight management [8]. The synergy of protein and fiber in pulses plays a role in stabilizing blood sugar levels, an important factor in diabetes management [8]. Cereal grains such as wheat and rice exhibit a lower protein content, typically 8% to 15%, in contrast to pulses and soybeans. Despite their low protein levels, cereals provide a substantial carbohydrate content, a primary energy source [12]. They are also important sources of essential vitamins and minerals, thereby enhancing the overall nutritional value. Pseudo-cereal grains such as quinoa and amaranth contain a protein content generally ranging from 12 to 18%, which matches or exceeds that of cereals, and features a balanced amino acid profile. Significantly, pseudo-cereals are gluten-free, making them suitable for individuals with gluten sensitivities or celiac disease [12]. 

Soy protein is predominantly composed of storage proteins, which are ~90% of its content [11]. The structural characteristics of these storage proteins critically influence the physical, chemical, and functional attributes of soy proteins. According to different sedimentation coefficients (Svedberg Units or S) after centrifugation, glycinin (11S) and β-conglycinin (7S) are the primary components, together accounting for over 70% of soybean storage protein, and thus significantly impact soy protein functionality [11]. Glycinin, or 11S, is an oligomeric protein formed by linking acidic and basic peptides via disulfide bonds. In contrast, 7S is a trimer comprising three subunits (α, α’, and β) bound through non-covalent interactions [34]. Notably, these protein components are susceptible to denaturation under harsh processing conditions, such as alkali acid precipitation and high temperature drying, typical in industrial soy protein production [11]. 

Pulse proteins primarily consist of globulins and albumins, with prolamins and glutelins as minor constituents [6]. Globulins are divided based on their sedimentation coefficients into legumins (11S–12S globulins, 300–400 kDa) and vicilins (7S globulins, 150–180 kDa) [6]. Legumin exhibits a more rigid conformation, featuring an extensive quaternary structure and disulfide bridges, compared to vicilin. Vicilin subtypes include β-conglycinin, γ-conglycinin, and basic 7S globulin (Bg), each differing in functional properties [35]. Legumin is rich in sulfur-containing amino acids (AAs) and arginine, while vicilin has higher levels of isoleucine, leucine, phenylalanine, and lysine [8]. Both 11S and 7S are salt soluble, whereas albumins (2S) are water soluble. Albumins encompass enzymes, protease inhibitors, amylase inhibitors, and lectins, with molecular weights (Mws) ranging between 5 kDa and 80 kDa, and generally have higher cysteine and methionine concentrations than pulse globulins. Prolamins, minor proteins in pulses, are rich in proline and glutamine and alcohol soluble [8]. Glutelin, soluble in dilute acid or alkali detergents, has higher methionine and cystine levels compared to globulins [8]. The potential of enhancing the glutelin content in pulses through breeding to improve protein quality has been suggested [36].

Cereal protein content and composition vary by cereal type, generally classified by solubility using the sequential Osborne extraction method that isolates albumins, globulins, gliadins, and then glutenin [1]. In prevalent cereals, rice bran protein is notable for its highest albumin and globulin levels. Rice proteins are characterized by significant gliadin content, while wheat and sorghum proteins are rich in gluten. Despite its low protein content, rice is considered a high-quality protein source [37]. In brown rice, albumin, globulin, gliadin, and glutenin comprise 5%~10%, 10%~15%, 70%~80%, and 1%~5% of total protein, respectively, with Mws of 10~200 kDa, 16~130 kDa, 7~12.6 kDa, and 19~90 kDa, respectively. The low gliadin content results in a high lysine concentration in rice compared to other cereals. Albumin is rich in histidine and threonine, while gliadin has high isoleucine, leucine, and phenylalanine levels [38]. Corn protein composition includes albumin, globulin, gliadin, and glutenin, constituting 5%~10%, 10%~15%, 50%~55%, and 20%~30% of the total protein, respectively. The Mw of albumin mostly ranges from 20 to 80 kDa, while gliadin and glutenin have Mws primarily at 22.513 kDa, 45.896 kDa, and 42.217 kDa, 32.129 kDa, respectively. Glutamine forms about a third of total amino acids in corn. Zein, a prolamin, is insoluble in water and anhydrous alcohols but soluble in 60% to 95% water–ethanol solutions, strong alkalis (pH >11), and certain organic solvents such as dodecane sodium sulfate [39]. Sorghum protein primarily includes gliadin and glutenin, constituting 60%~65% and 30%~35% of total protein, respectively, with albumin and globulin each contributing 1%~5% to total protein [39]. Albumin and globulin exhibit malleability with rough, porous surfaces and loose textures, while prolamins appear as spherical fine particles with a diameter of 0.4~2.0 μm. In contrast, gluten proteins are irregular blocks with smooth surfaces and dense textures. Albumin and globulin have a Mw distribution of 10 to 27 kDa. Gliadin typically has a Mw of 23 to 25 kDa, whereas glutelin ranges from 13 to 20 kDa [24]. The albumin and globulin in rice bran protein, with Mws of 10~200 kDa and 16~130 kDa, respectively, are physiologically and metabolically active proteins; its gliadin and glutenin are storage proteins with Mws of 7~12.6 kDa and 19~90 kDa, respectively. The amino acid composition of both albumin and globulin is well-balanced, with higher lysine and tryptophan levels compared to other cereals [25]. Wheat protein composition is complex. Albumin and globulin, primarily located in the aleurone layer, embryo, and seed coat, are metabolic or structural proteins, contributing 9% and 5% to total protein content. They are rich in lysine, tryptophan, and other essential amino acids, thereby enhancing their nutritional value. These proteins also encompass various physiologically active proteins that intricately regulate various metabolic activities in wheat development but have minimal influence on processing quality. In contrast, gliadin and glutenin, present in the endosperm, constitute 40% and 46% of total protein, respectively [12], and are categorized as storage proteins or glutenin. 

Most pseudo-cereals used in breadmaking belong to the dicotyledon class. Similar to rice, they are rich in starch, and predominantly consist of globulins [40]. Pseudo-cereals, compared to traditional cereals, contain higher levels of proteins rich in arginine, tryptophan, lysine, and histidine, featuring 7S and 11S globulins [41]. Typical 11S globulins exhibit a hexametric structure, with a Mw between 250 and 400 kDa, comprising acidic and basic chains linked by a single disulfide bond [42]. In contrast, 7S globulins form a trimeric configuration with a Mw ranging from 150 to 190 kDa and lack disulfide bonds. Albumins have Mws between 8 and 15 kDa, and consist of small and large subunits connected by two disulfide bonds [42]. According to Taylor et al. [43], the primary storage proteins in pseudo-cereals closely resemble legume proteins, including 2S albumin, 7S, and 11S globulin storage proteins. Pseudo-cereals such as amaranth, quinoa, and buckwheat are used in gluten-free bread formulations, contributing to improved shelf life when their flours are added to the dough. Moreover, pseudo-cereals have a higher lipid content than most cereals, especially in unsaturated lipids [44]. Linoleic acid is the dominant unsaturated fatty acid in these grains, comprising 52% in quinoa, 46.3% in amaranth, and 36.4% of buckwheat, followed by oleic acid at 33.9% in buckwheat, 24% in quinoa, and 23.1% in amaranth [44]. The increased consumption of n-3 fatty acids has been linked to reduced biomarkers for various degenerative diseases, such as cardiovascular disease, cancer, osteoporosis, inflammation, etc. [44]. In summary, pseudo-cereal proteins not only offer a considerable nutritional profile but also exhibit a low allergenic potential, making them a promising sustainable alternative in protein sources.

## 3. Modification Techniques of Soy, Pulse, Cereal, and Pseudo-Cereal Proteins

**Figure 2:**
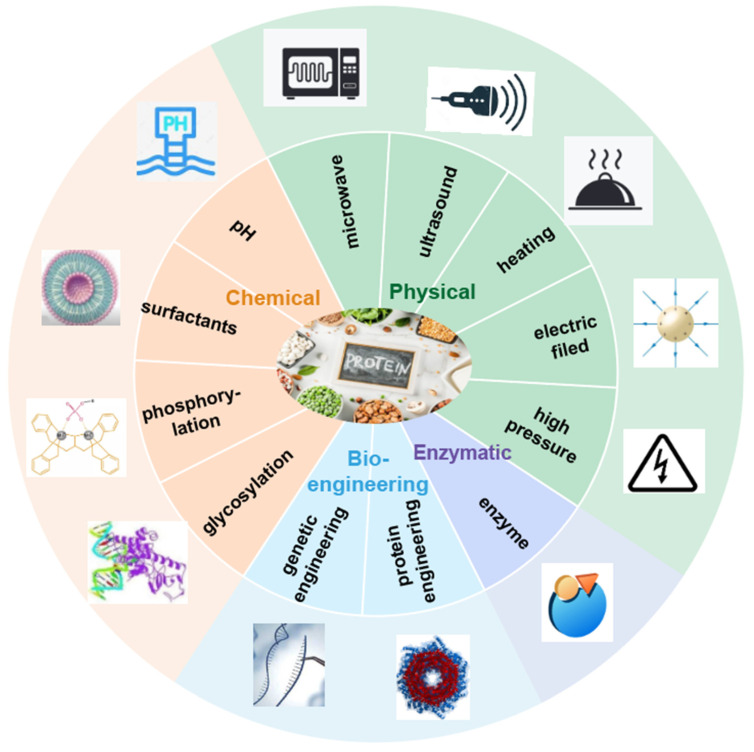
Protein modification methods.

Protein modification pertains to the alteration of amino acid residues and polypeptide chains, leading to changes in protein spatial structure and properties to enhance their functionalities [3]. The techniques are broadly classified into physical, chemical, enzymatic, combined, and bioengineering methods (Figure 2) [45]. Among these, bioengineering methods, including genetic engineering and protein engineering, raise potential safety concerns. In this review, the other methods are highlighted due to their established applications in foods (Table 2). 

### 3.1. Physical Modification

Physical modification techniques induce protein aggregation or structural changes via extrusion, heating, ultrasonication, pulsed electric field, high-pressure homogenization, and microwave without altering the primary structure [47]. In summary, physical modification methods are valued for incurring minimal nutritional loss, requiring short processing times, and being cost-effective.

Heat treatment induces the exposure of protein hydrophobic groups and changes in tertiary structure, and increased temperatures result in the formation of disulfide bonds (S-S), salt bridges, and intensified hydrophobic interactions among protein molecules, which lead to alterations in protein functional properties such as protein solubility [46]. For instance, Zhao et al. [46] observed a significant reduction in the solubility of rice glutelin after being exposed to 80 °C for 30 min. Similarly, Yin et al. [53] reported a decrease in the solubility of cannabis protein isolates after heat treatment at 95 °C for 10 min. It indicates that mild heat treatment increases disulfide bond formation within the protein, resulting in the formation of insoluble aggregates. On the other hand, extreme heat treatment can disrupt disulfide bonds and thus enhance protein solubility. For example, Zhao et al. [46] reported a threefold increase in the solubility of rice glutelin after exposure to 100 °C for 60 min. This effect was also observed in soy and pulse proteins [54]. Other functional properties such as color, aroma, and flavor can be changed due to heating [46]. Notably, heating was found to reduce the raw or beany taste in soy protein solutions and improve their digestibility [27]. Specific heat treatments, such as steam cooking, enhance nutritional quality by increasing protein digestibility and may induce caramelization, adding sweet and caramel-like flavors [27]. However, excessive caramelization may cause bitterness, underscoring the need for careful consideration of protein ingredient type, heating duration and temperature, and environmental conditions [18].

Ultrasound, as a novel, non-thermal, and eco-friendly technology, has attracted attention for protein modification. This technology utilizes sound waves exceeding the human auditory threshold of 20 kHz, which predominantly leverage cavitation, a rapid oscillation between compression and expansion within a confined local area in a material, to mechanically disrupt macromolecular bonds through tension and collapse [37]. Numerous studies highlight that the ultrasound duration and frequency combination, and power level, significantly influence protein structural integrity [48]. The solubility of soy protein isolates had an initial increase followed by a decrease with extended sonication [48]. Xiong et al. [55] reported that pea protein ultrasonically treated at 20 kHz formed more stable emulsions than untreated samples, for which ultrasound reduced particle size and increased surface hydrophobicity, thereby reducing oil–water interfacial tension, and improving foaming properties. Wang et al. [56] also reported enhanced antioxidant properties in pea protein extracted with ultrasound. Moreover, ultrasound is effective in modifying other legume proteins, such as those from lentils, broad beans, and peanut beans, as well as enhancing the functionality of cereal and pseudo-cereal proteins [57,58]. Despite these advancements, ultrasound application in protein modification is predominantly at laboratory scale, with scaling to pilot or industrial levels presenting a key challenge.

Pulsed electric field (PEF) modification, which employs high electric field intensities, short pulse widths, and increased pulse frequencies, induces changes in the microenvironment and molecular conformation of protein molecules. Taha et al. [59] observed that soy protein treated with PEF at 30–50 kV/cm had reduced solubility. Similarly, reduced solubility was found in peas (from 23.2% to 17.2%), rice (from 16.4% to 9.2%), and gluten (from 25% to 22.4%) concentrates after moderate electric field strength treatment (1.65 kV/cm) [60]. PEF could cause a denaturation and aggregation of proteins, probably through the formation of hydrophobic interactions and S-S bonds [59]. Another possible reason is that PEF could facilitate the cross-linking of amino acid side chains, further diminishing protein solubility [59]. Similar results were also reported for sunflower protein [61]. However, Li et al. [49] found that PEF treatment at up to 30 kV/cm using bipolar waveforms improved the solubility of soy protein isolates, with higher field strengths causing only a slight decline in solubility. An increase in canola protein solubility after PEF treatment at 35 kV/cm for 8 μs was observed [61]. Thus, waveform variations and specific protein type influence solubility differently. The potential of PEF to improve the flavor profile of plant proteins has been highlighted. However, research into the effect of PEF on plant protein digestibility and anti-nutritional factors is limited, indicating a valuable direction for future investigation.

### 3.2. Chemical Modification 

The chemical modification of proteins involves altering functional groups within polypeptide chains, such as carboxyl (-COOH), hydroxyl (-OH), sulfhydryl (-SH), and amino (-NH_3_) groups [62]; during such processes, the intramolecular electrostatic interactions, molecular structure, and hydrophobicity are modified, which consequently modify the functional properties of proteins, such as solubility, foaming capacity, emulsifying properties, gel formation, and thermal stability. Techniques for chemically modifying food proteins include acid–base modification, glycosylation, phosphorylation, chemical cross-linking, and surfactant use [62].

Acid–base treatment is a prevalent method for protein modification, leveraging pH adjustments far from the protein’s isoelectric point to enhance solubility and various functionalities. When protein surfaces acquire charges in such environments, ion-dipole interactions between charged amino acids and water molecules are facilitated, increasing solubility. This process also produces uniform repulsion among similarly charged protein surfaces, promoting the unfolding of protein molecules [63]. Extreme pH conditions can disrupt intermolecular interactions and cleave disulfide bonds, altering the protein’s secondary and tertiary structures and increasing molecular flexibility. Jiang et al. [63] observed that an alkaline pH shift to a pH of 12 increased the surface activity of pea protein with enhanced adsorption at the emulsion interface and improved emulsion oxidative stability, which might stem from the more extended protein structure with charged groups exposed. Jiang et al. [50] also observed that pH shift treatment significantly enhanced the gel strength of soy and pea proteins, which was because that pH shift could break the existing disulfide bonds and facilitate the formation of new ones. Although pH shift processes are typically conducted at room temperature, research indicates that proteins in highly acidic or alkaline conditions become more temperature sensitive. Liu et al. [54] adjusted the pH of soy protein to pH 1.5 at 50 or 60 °C and found that improved gelling properties but reduced solubility. On the other hand, Wang et al. [64] observed a higher solubility of hemp seed protein with higher pH shift temperature with a solubility peak at 97.5% when the pH was shifted for 60 min at 80 °C; however, excessive temperatures or prolonged exposure times can lead to the loss of amino acids. Acid–base treatment is also used in modifying cereal and pseudo-cereal proteins. For example, it is widely used in wheat dough systems to modify dough rheology by increasing water-holding capacity with the aim of increasing shelf life and improving product aroma profile [43]. Another study used strong acid or alkali (0.5–2 M HCl and NaOH), coupled with or without heat, to isolate Z19 α-zein, which led to the partial unfolding of the protein and improved emulsifying properties [65]. Similarly, when isolated oat globulins were treated with NaOH (pH 9.7) at 25, 37, and 55 °C, insoluble aggregates formed at 25 and 37 °C and soluble aggregates at 55 °C [43]. Amaranth proteins showed conformational changes and partial denaturation at extreme pH levels with surface hydrophobicity reduced at acidic pH compared to alkaline pH [66]. Despite the benefits presented with acid–base modification, it is important to acknowledge the potential drawbacks such as the potential for undesirable chemical residues that compromise the quality and safety of modified plant proteins. Furthermore, essential nutrients may be lost, diminishing the nutritional value and the health advantages. Ongoing efforts by researchers and manufacturers aim to refine these processes to meet consumer expectations and regulatory requirements.

Glycosylation refers to the attachment of carbohydrate moieties to lysine residues or the N-terminus of proteins [62], which commonly occurs during the Maillard reaction. This process can be implemented via dry- and moist-heat methods. In dry-heat glycosylation, a mixture of protein and polysaccharide in aqueous solution is dehydrated before being heated under specific conditions, with the process ceasing as the mixture cools to ambient temperature. Although this method yields a higher grafting rate, its lengthy duration limits. The moist-heat technique involves heating a pre-mixed solution of proteins and polysaccharides at a set temperature, offering a quicker reaction time but lower grafting efficiency, and an increase in by-products [67]. 

Glycosylated food products must enhance functionality without significantly altering the color or flavor, which underscores precise control over the Maillard reaction to prevent unwanted sensory changes [67]. Ma et al. [68] used a dry-heat grafting reaction to improve soy proteins’ emulsifying properties with citrus and apple pectin. Wang et al. [69] enhanced emulsion stability by grafting deamidated wheat gluten with maltodextrin, to create a product more resilient against pH and ionic strength changes. Zha et al. [70] treated pea protein isolates with gum arabic at 60 °C that enhanced their solubility and emulsification capacity. A recent study reported glycosylation for modifying quinoa protein that improved its specific surface area and absorption capacity as well as the anti-inflammatory and anti-proliferative activities. Ongoing research is crucial for a comprehensive understanding of glycosylation’s effects on pseudo-cereal proteins. While the glycosylation reaction is efficient, it can generate by-products that compromise the safety of the final product, including lanthionine, acrylamide, furan, pyruvaldehyde, and 5-hydroxymethylfurfural (HMF) [51]. These toxic compounds could disrupt normal protein structures and functions, undermining food safety and nutritional quality. They may also be associated with an increased risk of cancer. Therefore, it is crucial to control glycosylation process parameters, such as temperature, pH, and reactant concentrations [71], as well as perform thorough safety assessments of the final products. These findings highlight the need for careful consideration when using glycosylation.

Esterification and phosphorylation are also important chemical methods for modifying food proteins [72]. Esterification with different alcohols blocks free carboxyl groups, thereby increasing the net positive charge and basicity of the modified proteins. This process can significantly enhance the functional properties of proteins, such as their emulsifying capacity, solubility, and stability. For example, the solubility of two pulse proteins (broad bean and chickpea) improved within the pH range of 2.0 to 5.0 after esterification [73]. Additionally, the emulsification and foaming properties of these esterified pulse proteins in the acidic pH range of 2.0 to 6.0 surpassed those of the native proteins. Wang et al. [52] found that altering the protein isoelectric point (pI) through esterification could be a feasible way to enhance these properties. All esterified-protein nano-emulsion samples exhibited small mean particle sizes, good stability, and an antibacterial-inhibitory effect under weakly acidic conditions (pH 5.0). On the other hand, phosphorylation involves the covalent attachment of phosphoryl groups (PO_3_^−^) to specific reactive amino acid residues (-NH, -OH, or -SH) in the protein molecule [62]. This modification increases protein hydrophilicity by deprotonating and adding negative surface charges, thereby enhancing solubility. Liu et al. [74] reported that the solubility, emulsifying properties, foaming properties, and oil absorption capacity of pea protein isolate significantly improved after phosphorylation. However, chemically phosphorylated proteins have not been readily accepted by health-conscious consumers and are less desirable for food applications due to harsh reaction conditions and nonspecific chemical reagents. Additionally, the apparent molecular weight and thiol content of phosphorylated soy protein isolate increased with the degree of phosphorylation, which negatively affected gastrointestinal digestibility, particularly gastric digestibility [75]. Therefore, to safely use phosphorylated proteins as food ingredients, the potential toxicity of residues introduced into legume proteins must be seriously considered [72]. Experiments with mammals are essential to evaluate the toxicity and physiological functions of phosphorylated proteins.

### 3.3. Enzymatic Modification 

Enzymatic modification employs enzymes to modify the amino acid residues and polypeptide chains of proteins, enhancing their structural and functional characteristics. Mo et al. [76] utilized tyrosinase to hydrolyze soy protein to improve its viscosity and hydrophobicity. Liu et al. [77] used transglutaminase pretreatment on soy protein to produce a protein-based film with superior tensile strength and water-holding capacity. Peptides hydrolyzed from pea protein exhibited potential therapeutic benefits for kidney diseases as they could inhibit *Staphylococcus aureus* and *Escherichia coli* and act as inhibitors for trypsin and chymotrypsin [78]. Daliri et al. [79] employed pancreatin to hydrolyze quinoa protein concentrate and enhanced its solubility, emulsification, and foaming properties. Kamal et al. [80] used bromelain, chymotrypsin, and pronase to hydrolyze amaranth protein to produce bioactive peptides with inhibitory effects against enzymes associated with hypertension and diabetes. In summary, enzymatic hydrolysis emerges as a potent method for generating plant protein-derived peptides with a range of biological functions, including antioxidant, antimicrobial, and antihypertensive effects. Plant proteins and peptides are increasingly recognized as valuable components for developing novel food products. Continued research is essential to assess the influence of plant proteins on the quality of final products and food formulations. Despite the advantages, enzymatically modified proteins may face challenges such as high enzyme costs and the development of an undesirable bitter taste [62]. These challenges could limit the broader application of enzymatic modification techniques. 

### 3.4. Combined Modification

Combined modification, integrating multiple techniques to modify proteins, leverages the strengths while mitigating limitations of each method to enhance protein functionality, which cost-effectively optimizes outcomes. Liang et al. [81] combined heat treatment and pepsin to enhance the foaming and emulsifying properties of soy protein isolate. Wang et al. [64] applied pH shift and heat treatment to improve hemp seed protein by altering its structure. Bessada et al. [8] used ultrasonication with pH shift to increase pea protein solubility, possibly by disrupting disulfide bonds and exposing hydrophobic groups. Figueroa et al. [82] investigated the effects of pH-shifting and ultrasonication on the physicochemical characteristics of amaranth protein, finding that combined modification enhanced protein solubility and digestibility. Urbizo et al. [33] prepared chia protein hydrolysates using ultrasound treatment followed by microwave-assisted hydrolysis, and they exhibited significantly higher antioxidant activity, alongside potential antidiabetic and antihypertensive effects. 

### 3.5. Safety Issues of the Modification Technologies

Ensuring the safety of modified proteins is crucial for their usability in the food industry. Although the above modification techniques can enhance protein functionalities to a certain extent, they pose several potential safety concerns. For example, excessive or improperly controlled heat treatments can generate undesirable risk factors, such as advanced glycation end products (AGEs) and heterocyclic amines (HAs) [83]. AGEs are harmful compounds formed through non-enzymatic reactions involving the polymerization, condensation, and other interactions between free amino groups of proteins and the active carbonyl groups of reducing sugars or intermediate carbonyl compounds from the Maillard reaction [83]. AGEs have been linked to chronic diseases, including diabetes and cardiovascular disorders, while HAs, produced at high temperatures, are potential carcinogens that can increase cancer risk [83]. Therefore, optimizing heating conditions is essential to balance functional improvements while minimizing the formation of such harmful compounds. Regarding ultrasound and PEF treatments, the heat generated by ultrasound can potentially denature heat-sensitive compounds, presenting a significant limitation. Additionally, there is a risk of contamination from metals detaching from the probe [37]. In the PEF process, the materials used as electrodes, such as titanium and platinum, could migrate into the protein solution [84]. Thus, regular monitoring and maintenance of ultrasound and PEF systems are necessary to ensure the safety and efficacy of these physical methods.

Similar to heat treatment, the glycosylation reaction can produce by-products that compromise the safety of the final product, including lanthionine, acrylamide, furan, pyruvaldehyde, and 5-hydroxymethylfurfural (HMF). During glycosylation, the side chains of proteins (e.g., serine and cysteine) can undergo the elimination of H_2_O or H_2_S, resulting in dehydroalanine (DHA), which can then react with amino or thiol groups in lysine and cysteine to form protein cross-links such as lysinoalanine (LAL) and lanthionine (LAN) [85]. Poojary et al. [85] found that these cross-links are often detected in processed foods (protein concentrates, meat, and dairy products) and may impair protein digestibility and nutritional quality. The use of chemical agents in acid–base and other chemical treatments can leave behind residues that are toxic or detrimental to the nutritional quality of the protein. If not properly controlled, these methods can introduce allergens or trigger immunogenic responses [86]. Thus, the potential for residual chemicals, the formation of toxic by-products, and the introduction of allergenic or immunogenic compounds are significant safety concerns in the chemical modification process. In conclusion, comprehensive safety assessments, including toxicological testing, allergenicity evaluations, and rigorous quality control measures, are necessary to ensure the safety of both physically and chemically modified proteins.

## 4. Bioactive Properties of Soy, Pulse, Cereal, and Pseudo-Cereal Proteins

**Figure 3:**
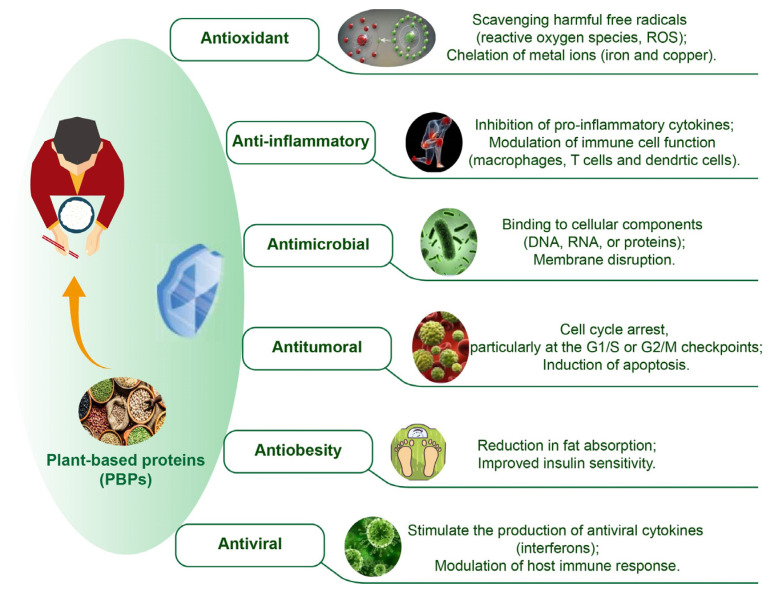
The bioactive properties and potential mechanisms of plant-based proteins.

The rising interest in plant proteins is largely attributed to their bioactivity and health-promoting potential. These proteins are rich in phytochemicals, such as polyphenols, flavonoids, and antioxidants that provide anti-inflammatory, antioxidant, and anti-carcinogenic properties. Furthermore, they contain or could be produced into bioactive peptides that play roles in regulating blood pressure, modulating immune responses, and inhibiting enzymes. Such properties not only enhance health and wellness but also facilitate the development of functional foods and nutraceuticals aimed at specific health benefits. The ongoing research on the bioactive potential of plant proteins signals a promising direction for food innovation in nutrition and preventive health care (Figure 3).

### 4.1. Antioxidant Activity

Free radicals, by-products of metabolic processes, can lead to oxidative stress when the balance between reactive species production and antioxidant defense is disrupted [8], and this process underpins various pathologies. Dietary compounds, such as proteins, peptides, amino acids, and phenolics are essential for strengthening the body’s antioxidant system [8,87]. Pulses and other plants are rich in major phenolics like tannins, phenolic acids (e.g., gallic, syringic, chlorogenic, and caffeic acids), flavonoids (e.g., catechin and epicatechin), and anthocyanins [87]. Thus, plant-based phenolics play a critical role in enhancing antioxidant defense.

On the other hand, hydrophobic amino acids are crucial for scavenging free radicals, whereas polar amino acids excel in reducing and chelating metal ions. Hydrophobic residues like tyrosine, tryptophan, phenylalanine, leucine, isoleucine, and alanine, act as hydrogen donors in the peroxidation of aromatic side chains, enhancing antioxidant defenses [26]. For example, glycinin (11S) could scavenge free radicals, largely due to its high content of amino acids such as tyrosine, thereby protecting cells from oxidative damage [88]. Bahareh et al. [89] observed that the basic and aromatic amino acid residues found in phaseolin peptides significantly increased antioxidant capacity, as histidine, alongside aspartic and glutamic acids, acts as a metal chelator, mitigating prooxidant effects. In addition, the proteolytic digestion of soybean 7S protein produces a pentapeptide (Leu–Leu–Pro–His–His) that exhibits antioxidant activity [88]. Moreover, peptides derived from rice bran protein via papain and trypsin hydrolysis exhibited antioxidant properties, especially when a hydrophobic amino acid is positioned near the N or C terminus [90]. The role of serine and threonine residues in enhancing the antioxidant potential of millet protein hydrolysates was studied [91]. Antioxidant activities of pseudo-cereal proteins, such as those from quinoa and amaranth seed, were also explored [92]. These antioxidant activities are crucial for mitigating oxidative stress-related diseases, such as cardiovascular diseases, cancer, and neurodegenerative disorders. 

### 4.2. Antimicrobial Activity

Several plant proteins have been recognized as plant defense mechanisms with antibacterial properties in inhibiting the growth of various pathogens, including bacteria, fungi, and viruses. For example, lupin protein hydrolysates exhibited antibacterial activity against both Gram-positive and Gram-negative bacteria in food systems, indicating their applications as natural food preservatives [93]. Moreover, various pulse proteins and peptides exhibit antimicrobial potential against several foodborne pathogens. For instance, chickpea-methylated proteins are effective against *Listeria monocytogenes*, and chickpea proteins and pea peptides are active against *Salmonella* spp. [94]. Lentil lectins and common pea peptide fractions showed antimicrobial potential against *Escherichia coli*, and *Staphylococcus aureus* [94]. *Vicia faba* peptides exhibited inhibitory effects against nosocomial pathogens like *Pseudomonas aeruginosa* [95]. Proteins and peptides derived from cowpea and lupine had antimicrobial efficacy against a broad spectrum of bacteria to mitigate microbial proliferation in meat products [96]. The minimum inhibitory concentrations (MICs) for cowpea proteins and lupine protein hydrolysates were comparable with soybean proteins and peptides and some medicinal plant extracts. These proteins induce bacterial cell deformation and lysis, underlining a potent antibacterial mechanism [8]. The antimicrobial potential of pseudo-cereals like buckwheat, quinoa, and amaranth has also been investigated. Mudgil et al. [97] found that amaranth and quinoa protein isolates showed a significant inhibitory impact on *Staphylococcus aureus* and *Escherichia coli*. Therefore, antimicrobial plant proteins are sustainable alternatives to traditional antimicrobials and have potential for novel therapeutic and agricultural applications.

### 4.3. Antitumoral Activity

Proteins and peptides from legumes, in particular, have gained interest due to their genotoxicity-free nature, genotype specificity, and potential as adjuvant cancer therapies [8]. For example, β-conglycinin could inhibit the proliferation of cancer cells by inducing apoptosis and causing cell cycle arrest. Its bioactive peptides, released during digestion, can disrupt cancer cell signaling pathways, thereby reducing tumor growth and metastasis [98]. Peptides from pulses have shown anti-gastrointestinal cancer potential, peptides from common beans have impacted human colorectal cell lines, and the pulse proteins from common bean, fava bean, lupin, lentils, chickpea, and cowpea are associated with anti-proliferative effects and reduced cell migration [8]. Similarly, hydrophobic peptides from glycinin exhibit significant anti-cancer activity by inducing apoptosis in cancer cells and inhibiting cell migration [99]. An Arg–Gly–Asp tripeptide has been identified as the adhesion region of soy lunasin to cells, adhesion being a prerequisite for its anticarcinogenic properties [88]. Furthermore, whole cereal consumption is linked to lower risks of chronic degenerative diseases. Ortiz-Martinez et al. [100] reported anti-cancer effects of maize peptides in HepG2 cells. Vilcacundo et al. [101] explored anti-cancer properties of quinoa protein hydrolysates in colon cancer cells. Thus, exploring antitumoral plant proteins opens a promising avenue for novel natural compounds in cancer treatment innovation.

### 4.4. Other Activities

Plant proteins exhibit bioactive properties, such as anti-hypertensive, anti-thrombotic, and anti-inflammatory activities [8]. Protein hydrolysates from defatted flours of common beans, peas, chickpeas, lentils, and lupin, via pepsin digestion, showed anti-hypertensive effects. Among them, lupin peptides, primarily produced from α and β-conglutin, exhibited the highest efficiency [102]. Rice globulin peptides, such as Tyr-Tyr-Gly-Gly-Glu-Gly-Ser-Ser-Ser-Glu-Gln-Gly and Ser-Glu-Ser-Glu-Met, were associated with atherosclerosis inhibition through antiapoptotic, antioxidant, and anti-inflammatory mechanisms [103]. Wheat peptides and millet protein hydrolysates exhibit anti-inflammatory potential, protecting against gastric damage in rats by reducing oxidative stress. The impact of cereal protein peptides in preventing type 1 diabetes also underscores their anti-inflammatory efficacy [104]. Furthermore, β-conglycinin also exhibits anti-inflammatory properties, aiding in managing inflammatory conditions and promoting overall health. This 7S protein subunit is recognized for enhancing lipid profiles and mitigating obesity-related complications [105]. Ferreira et al. [106] found that the daily oral administration of β-conglycinin in the diet of hypercholesterolemic (HC) rats resulted in decreased total cholesterol (TC), low-density lipoprotein (LDL) cholesterol, and triglycerides (TG). Additionally, there was a significant reduction in cholesterol and TG levels in the liver. These findings showed that β-conglycinin is beneficial to cardiovascular health. Glycinin has been investigated for its cholesterol-lowering effects, as it can modulate lipid metabolism and reduce blood cholesterol levels [107]. 

## 5. Food Applications of Soy, Pulse, Cereal, and Pseudo-Cereal Proteins

**Figure 4:**
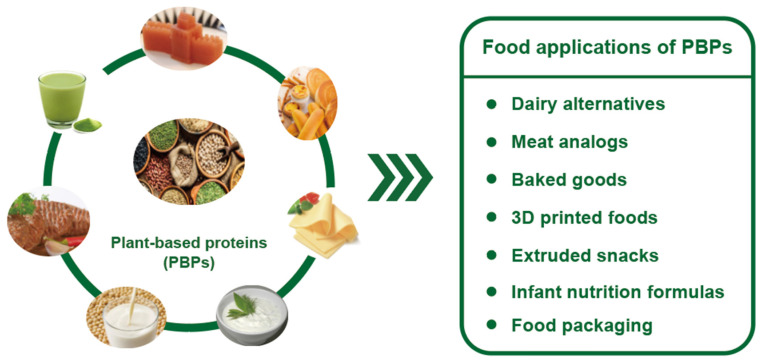
The commercial applications of plant-based proteins.

Plant proteins are used as a foundational ingredient to facilitate the creation of meat substitutes, dairy-free options, and plant-based snacks and beverages. Sourced from soybeans, peas, and rice, these proteins not only enhance the nutritional profile but also contribute significantly to the desired texture and flavor of foods. Plant proteins also meet consumer needs for nutrition, convenience, and diverse dietary preferences, including vegetarian and flexitarian diets. The potential applications of various plant proteins are shown in Figure 4.

### 5.1. Dairy Alternatives

Soy-based products are currently the most accessible milk alternatives with protein levels similar to cow’s milk, in contrast to almond milk and other popular options that typically contain less than 0.5% protein [18]. This underscores the demand for new protein sources in milk substitutes. Pulses, offering substantial protein content, are emerging as viable alternatives for developing milk substitutes, with pea protein-based products leading this innovation [108]. Ongoing research also explores the suitability of fava beans, chickpeas, and other pulses in crafting alternatives to traditional dairy milk, and the research found that chickpea-based beverages achieved sensory acceptance levels comparable to those of soy-based beverages, despite chickpeas having lower protein and higher carbohydrate contents [108]. To successfully integrate into milk and dairy alternative products, pulse proteins must demonstrate outstanding functional properties such as solubility and emulsification capabilities [18]. Additionally, the typically low oil content in pulses requires the addition of extra oil to match the fat levels of cow’s milk or soy beverages. Lupin is a promising substitute, favored for its high protein content and low starch levels [108]. A detailed evaluation of dairy alternative beverages made from pulses ranked their sensory attributes as follows: pea > lupin > lentil > chickpea > fava bean [108], and the study showed that although there were minor differences in color, the “beany” taste in chickpea and fava bean beverages notably reduced their acceptance.

Cereal proteins from grains such as oats, rice, and barley, offer a promising alternative to traditional milk due to their unique emulsifying and texturizing capabilities. Oat protein concentrates play a significant role in the formulation of several dairy-alternative beverages [109]. Oat milk is commercially available from brands like Oatly (Sweden), Pureharvest (Australia), Alpro (UK), Bioavena Drink (Italy), Simpli (Finland), Vitasoy (Hong Kong), and Pacific (USA). Oat beverages have cholesterol-lowering properties [18], making them a favorable option for health-conscious consumers. Despite its therapeutic benefits, oat milk has low calcium content, which highlights the need for fortification to match the nutritional value of cow’s milk [18]. In addition, rice-based beverages have emerged as alternatives for children with cow’s milk allergies [38]. Paul et al. [16] reported a robust annual growth rate of 15% for the global rice milk market from 2018 and 2023, and the major market participants include Vitasoy Australia Products Pvt Ltd., Campbell Soup Company, The Hain Celestial Group, Pureharvest, Panos Brands, LLC, and White Wave Foods. Although rice naturally contains the iron element, 85% of it is found in the bran [16], which is lost in rice milk production, highlighting the need for fortification. Rice bran milk has been developed to leverage the nutritional profile [16], and rice bran milk exhibits lower total soluble solids (TSS) values and viscosity than soy milk. Milk alternatives from pseudo-cereals, like amaranth, quinoa, and buckwheat, are available in some countries, but research on these milk alternatives remains limited [15]. This gap presents an opportunity for future studies to explore the potential of pseudo grain proteins for dairy alternative products.

### 5.2. Meat Analogs

Proteins from soy, peas, wheat, and lentils are utilized in crafting meat analogs that replicate the sensory and nutritional qualities of conventional meats [108]. Pulse proteins from mung bean, fava bean, peanut, velvet bean, and lupine have been investigated as effective substitutes in meat analogs [110]. For example, Beniwal et al. [110] reported that the hydrophilic and hydrophobic ratio of mung bean protein isolate was similar to that of soy protein, suggesting similar functional properties, such as water-holding and fat absorption capacity for achieving comparable texture in meat analogs. Žugčić et al. [111] studied the quality of beef patties with added pulse protein and determined their potential as an alternative to soy protein in that they exhibited similar taste and nutritional profiles. On the other hand, a study identified significant structural differences in commonly available meat alternatives, suggesting that proteins from sources like mung bean, pea, peanut, and lupin lack certain critical attributes for replicating a fibrous texture, despite the functional similarities of those proteins. Advanced processing techniques can transform plant proteins to mimic the fibrous texture of meat, enhancing the eating experience to allow for the creation of various meat substitutes, including burgers, sausages, nuggets, and meatballs [108]. Beniwal et al. [110] presented the feasibility of replicating the texture of ground meat products, such as chicken nuggets, burgers, and sausages, using textured vegetable protein, with an example showing that the use of pea or soy proteins combined with gluten was effective in mimicking chicken meat texture through shearing methods. Mokni Ghribi et al. [23] investigated the effects of adding chickpea protein concentrates in small amounts of 1.5% to 5% to cooked sausages, and found an improvement in lipid oxidation, color stability, antioxidant capacity, protein content, and consumer acceptance. Additionally, cereal proteins, especially wheat gluten, exhibit viscoelastic properties, making them excellent binders and texturizers in meat analog formulations [112]. Rice flour can be a fat substitute in meat products, particularly due to its water binding capacity. Research found that incorporating 4–6% rice flour into sausage meat products improved firmness while maintaining consumer acceptability [113]. Cereal proteins showed a more balanced amino acid profile when coupled with other plant-based proteins like peas or soy for mean analog production [108]. The integration of cereals with pulses is recognized as a high-quality protein source in meat analog development, including the use of pseudo grains such as quinoa and amaranth. Quinoa has shown promise in improving the water-holding capacity, reducing toughness, and enhancing sensory attributes of meat products [113], suggesting the potential for quinoa proteins to be used as a foundation for meat analogs. In contrast, the use of amaranth faces challenges due to its universally undesirable flavor [113], indicating the need for further research to refine its use in meat substitutes.

### 5.3. Baked Goods

Plant proteins sourced from peas, soy, wheat, and legumes offer functional and nutritional enhancements such as improved texture, structure, and moisture retention [108]. Hoehnel et al. [114] observed that substituting 15% of wheat flour with pea proteins reduced bread volume, while a similar replacement with fava bean concentrates yielded dough with medium gluten strength, indicating that incorporating pulse protein can enrich protein quality without significantly altering bread quality. Another research showed that introducing thermally modified cowpea proteins enhances water absorption and the acceptability of wheat bread [115], and that glycated and denatured cowpea proteins could replace up to 20% of whole egg content in sponge cake. Serventi et al. [116] successfully replaced a third of soy protein with chickpea proteins in bread and preserved bread texture. Plant proteins not only enrich the nutritional content of baked goods with essential amino acids, fiber, and micronutrients, but also offer innovative solutions for gluten-free options, such as pasta made from pulse proteins like peas and fava beans, in which pea protein contributes to a firmer texture, whereas fava bean protein improves water absorption, extrudability, and reduces cooking loss, making it different from semolina-based products [108]. Another study showed that fava bean protein in oat-based spaghetti increased chewiness and hardness, lowered the glycemic index, and enhanced protein digestibility [117].

### 5.4. 3D Printed Foods

3D printing technology has been used for the fabrication of foods with complex shapes [108], including meat analogs like beef, poultry, fish, and seafood. This technology leverages plant proteins from peas, soy, and lentils, to formulate printable materials, such as pastes or powders, to enable the precise layering and shaping of food structures [108]. For instance, Chen et al. [118] utilized 3D printing to produce steak-like foods using textured soy protein and defatted soy protein inks as a meat alternative, and the produced steak-like foods could meet different populations’ nutritional or energy needs as well as providing personalized sensory experiences. Oyinloye et al. [119] found that a blend of pea protein and sodium alginate in a 20:80 mass ratio led to satisfactory rheological and textural properties for 3D food printing. Qiu et al. [120] introduced air-heating printing to produce additive-free meat analogs incorporating rice protein, which modified the viscosity and water distribution, allowing them to be suitable for 3D printing. It is worth noting that the application of ancestral pseudo-cereals like quinoa, amaranth, and kaniwa in 3D printing remains unexplored [121], presenting an opportunity for future research considering their nutritional benefits. Furthermore, integrating artificial intelligence (AI) with 3D printing could optimize food ink formulations and automate printing procedures to contribute to the efficiency in this endeavor.

### 5.5. Other Food Applications 

Plant proteins are used in diverse food categories, including meat and seafood analogs, extruded snacks, meal-replacement drinks, soup mixes, and infant nutrition formulas [108]. For example, common bean, pea, and lentil flours, proteins, and concentrates have been integrated into low glycemic index foods, baby foods, and dietary supplements aimed at muscle growth, offering a plant-based alternative to whey products [8,108]. Clinical studies have demonstrated the benefits of lupin in flour or protein isolate form in reducing blood pressure for overweight individuals [8]. Innovations extend to food packaging, with a composite film made using kidney bean protein isolate and chitosan, which showed antimicrobial efficacy against *Bacillus subtilis* and *Staphylococcus aureus* [122]. Additionally, hydrolysates from peas, lentils, fava beans, and lupin flours are being explored as natural food preservatives, offering a sustainable alternative to extend the shelf life of food products [42]. These applications highlight the versatility of plant proteins and their role in promoting sustainability across the food sector. 

## 6. Conclusions and Future Perspectives

Plant-based proteins offer a sustainable solution to meet the increasing global food demand, and are primarily sourced from soybeans, pulses, cereals, and pseudo-cereals. Despite their potential, the functional properties of key proteins like 11S (glycinin) and 7S (β-conglycinin) are hindered by aggregation tendencies due to disulfide bonds and other interactions. Innovative methods such as enzymatic treatment and physical processing (e.g., ultrasonic) can enhance their functional qualities by modifying protein structures. These proteins excel in non-dairy alternatives, 3D food printing, and meat analogs, with additional benefits as antioxidants and antibacterial agents, fueling their popularity and development. However, challenges persist in mimicking the sensory qualities of animal products, with consumer acceptance hinging on the appearance, texture, and taste of plant-based alternatives. Issues with replicating the sensory profiles of meat and dairy, coupled with consumer concerns over added substances and the natural off-flavors of soybeans and pulses, underscore the need for focused research to overcome these hurdles and improve the marketability and acceptance of plant-based proteins.

## Figures and Tables

**Table 1:** Compositions (%, *w*/*w*) of major components of soybeans, pulses, cereals, and pseudocereals.

Classification	Type	Protein	Carbohydrate/Starch	Fiber (Crude)	Fat	Ash	Reference
Soybeans	Full fat soy flour	35–40	30–35	6–7	18–22	4–6	[13]
	Defatted soy flour	50–60	30–40	5–6	1–3	5–7	[14]
	Soy protein concentrate	65–70	20–30	3–5	1–2	6–8	[15]
	Soy protein isolate	90–95	1–3	<1	<1	<1	[16]
Pulses	Pea	14–31	55–72	3–20	1–4	2.3–3.7	[17]
	Lupin	32–55.3	4.5–47	14–55	5–15	2.6–5.09	[18]
	Chickpea	19–27	52–71	6–15	1–3	1.8–3.5	[18]
	Cowpea/black- eyed pea	23.6–33	37–52	2–5	1–2.1	2–5	[19]
	Lentil	23–31	42–72	7.0–23.0	1–3	2.1–3.2	[20]
	Yellow pea/field pea	21.4–25.9	49.3–56.6	11.6–18.4	0.8–2.1	1.1–2.8	[21]
	Black bean	22.9–23.2	60–65	3.4	1.6–3.4	4.6–5.0	[22]
	Faba bean	26.4–39.7	36.9–61.6	6.4–23.7	1.5–2.1	2.9–4.3	[23]
	Great Northern bean	20.8–23.6	45.5–47.2	15–20	1.3–1.7	3.8–4.4	[17]
	Lima bean	14.5–24.0	47.1–50.5	32.6–33.6	0.6–0.8	2.4–3.9	[22]
	Mung bean	25.8–27.5	52.2–52.8	2.2	1.6	2.9	[23]
	Navy bean	19–27	67–75	14–25	1.7–2.0	4.0–4.9	[18]
	Pinto bean	17.5–21.6	60–65	15–20	1.2–2.8	3.5–4.7	[22]
	Kidney bean	17–27	63–74	18–30	1–5	3.2–5.2	[18]
Cereals	Wheat	8.0–17.5	70–75	2–5	1–2	1.5–2	[24]
	Maize	8.8–11.9	70–80	2–4	2–5	1–2	[25]
	Rice	6.6–8.4	70–80	1.6–2.8	<2	0.4–0.7	[26]
	Oats	8.7–16.0	60–70	10–15	5–10	1.5–2.0	[27]
	Rye	8.0–17.7	60–70	10–15	<2	1.5–2.0	[27]
	Triticale	11.7–16.3	60–70	2–5	<2	1.5–2.0	[28]
	Teff	8.7–11.1	70–75	7–10	1–3	2–3	[27]
	Sorghum	7.0–15.0	70–75	6–12	2–4	1.5–2.0	[29]
	Millet	8.3–13.3	70–75	6–10	1–5	2–3	[27]
Pseudo-cereals	Buckwheat	21.6–25.3	70–75	8–12	2–4	2–3	[30]
	Amaranth	11.7–18.4	65–75	6–8	5–7	2–3	[27]
	Quinoa	13.0–14.0	60–70	2.5–4.0	4–7	2–3	[31]
	Fonio	9.0–10.0	70–75	2–4	1–2	2–3	[32]
	Chia	19.0–23.0	35–40	30–40	30–35	<5	[33]

**Table 2:** Description, advantages, and limitations of different protein modification methods.

Modification Category	Modification Method	Description	Advantages	Limitations	References
Physical	Heat treatment	➢ Rice glutelin was heated at 100 °C for 60 min (pH = 11.0). ➢ The solubility of rice glutelin was reached at 4.2 mg/mL.	Led to the denaturation of proteins, resulting in improved digestibility. Influence the texture and flavor of plant protein.	◆ Loss of nutritional value, such as vitamins and amino acids.	[46]
	Microwave	➢ Rice dreg protein (0.02 mg/mL, 50 mL) and sodium alginate combined and treated by intermittent microwave heating. ➢ Obtained the highest solubility at pH = 10.0 (90.97%).	Ease of use. Low solvent volume. High protein solubility. High protein digestibility. High potential for scale-up.	◆ Expensive equipment. ◆ Dielectric properties of the solid material to absorb microwaves. ◆ Microwave-induced radicals can oxidize protein, affecting the structure and functions of the protein.	[47]
	Ultrasound	➢ Soy protein isolate was treated by energy-gathered ultrasound power density of 100 W/L for 10, 20, 30, 40, and 60 min. ➢ The highest solubility was found at 30 min. ➢ The emulsion activity was increased by 11.8% as compared to that of control (*p* ≤ 0.05) at 30 min.	Low environmental burden. Less denaturation of protein due to the low operating temperature and temperature rise. Ultrasound improved physical, chemical, functional, and nutritional modifications of protein isolate.	◆ High energy consumption. ◆ High capital investment. ◆ Ultrasound can be used in pilot scale only up to date. ◆ Long-time use of the probe can lead to erosion of the metal tip into the protein solution; thus, a metal part can be present in the protein solution.	[48]
	Pulsed electric field (PEF)	➢ Using a PEF system with bipolar waveforms improved the solubility of soy protein isolates (30 kV/cm). ➢ PEF at strengths exceeding 30 kV/cm led to a marginal reduction in protein solubility.	Energy consumption is low. PEF is applied for a very short time (10^−4^ and 10^−2^ s), thus there is little heating effect on the sample (ΔT < 10 °C). Environmentally friendly technique.	◆ The efficiency of the method depends on electric field strength and electrode gap. ◆ High cost of the equipment, which implies a high initial investment. ◆ Commercial PEF units are not widely available in many regions worldwide.	[49]
Chemical	Acid–base treatment	➢ Pea protein solution (20 mg/mL, pH 7.0) was titrated to pH 12 with 2 M NaOH at 20 °C for 1 h. ➢ The alkaline pH shift (pH = 12) increased the surface activity by 52%.	Advanced protein solubility and functionality. Controlled gelation, leading to the formation of gels with specific textures and structures.	◆ The formation of undesirable compounds or flavors. ◆ Adds complexity to the industry process and require careful control of pH conditions.	[50]
	Glycosylation	➢ Pea protein isolates were mixed with gum arabic at a ratio of 1:4 (60 °C). ➢ The conjugation of PPI with GA enhanced solubility and emulsification capacity.	Increase protein stability, protecting them from proteolysis and degradation. Help proteins evade recognition by the immune system, reducing the likelihood of immune responses.	◆ Complex and expensive. ◆ Requiring sophisticated techniques and technologies.	[51]
Enzymatic	Enzymatic methods	➢ Quinoa protein concentrate was dissolved in 0.01 mol/L phosphate buffer (pH = 7.4) for 30 min and a pancreatin enzyme solution (in 0.01 mol/L phosphate buffer) was added to react at 40 °C for 4 h. ➢ The solubility, emulsifying, and foaming activities of the final hydrolysate surpassed those of the native protein.	Reduced solvent usage with lower energy consumption. An environmentally friendly green processing technique. Amenable to scale up.	◆ The expensive price of the enzyme. ◆ Requires an optimization process to minimize enzyme dosage to achieve a high recovery yield. ◆ Variations in enzyme activity between batches may have certain adverse effects.	[52]

## Data Availability

No new data were created or analyzed in this study. Data sharing is not applicable to this article.

## List of Abbreviations

GHG	Greenhouse gas
LDL	Low-density lipoprotein
11S	Glycinin
7S	β-conglycinin
AAs	Amino acids
2S	Albumins
MM	Molecular masses
S-S	Disulfide bonds
PEF	Pulsed electric field
MIC	Minimum inhibitory concentration
QPHs	Quinoa protein hydrolysates
OPCs	Oat protein concentrates
CAGR	Compound annual growth rate
RBM	Rice bran milk
TSS	Total soluble solids
MBPI	Mung bean protein isolate
WHC	Water-holding capacity
FAC	Fat absorption capacity
TVP	Textured vegetable protein
